# WHEN YOU GET TO A FORK IN THE ROAD, TAKE IT: SHOULD STATES FOLLOW YOGI'S ADVICE ON MANAGED LONG-TERM SERVICES?

**DOI:** 10.1093/geroni/igac059.746

**Published:** 2022-12-20

**Authors:** Robert Applebaum

**Affiliations:** Miami University, Oxford, Ohio, United States

## Abstract

Today’s Medicaid challenges, coupled with the baby boom demographics, have every state in the nation recognizing the need to do something different in their Medicaid programs. Although achieving a better balance between institutional and home and community-based services has been an important reform in many states, it does not appear to be enough to create a working system. Medicaid managed long-term care and efforts to integrate Medicare and Medicaid is a growing option. Designed to control the acute and long-term care costs of older people and individuals with disability, the approach also is directed at linking the two disparate systems. A review of the array of studies examining this area shows mixed results, despite the popularity of this option at the state level. This paper introduces the evaluation of Ohio’s MyCare integrated care demonstration, raising questions about the important elements of these initiatives for policy makers, providers, and consumers.

